# Comparison of Two Artificial Liver Treatments for Acute Liver Failure: A Retrospective Observational Study

**DOI:** 10.1111/nicc.70326

**Published:** 2026-02-07

**Authors:** Xiulian Wu, Dongxia Wu, Xiaoqing Sun, Wenhui Wang, Jiayuan Wei, Xin Liu, Yanmei Gu

**Affiliations:** ^1^ Department of Critical Care Medicine, Beijing You'an Hospital Affiliated to Capital Medical University Beijing China; ^2^ Department of Infection and Immunology, Beijing You'an Hospital Affiliated to Capital Medical University Beijing China

**Keywords:** adverse reactions, dual plasma molecular adsorption system, efficacy, liver failure, plasma exchange

## Abstract

**Background:**

Liver failure can lead to severe disorders in the body's metabolism, detoxification, synthesis and other functions, with a high mortality rate of 60%–90%. The dual plasma molecular adsorption system (DPMAS) is a new artificial liver technology that has received much attention in recent years. However, it cannot provide coagulation factors, albumin or other substances during the treatment process and may even consume albumin during the adsorption process. The DPMAS typically needs to be used in combination with plasma exchange (PE). Few studies have compared PE alone and DPMAS + PE.

**Aim:**

This study aimed to explore the clinical efficacy and nursing methods of different artificial liver treatments for patients with acute drug‐induced liver injury.

**Study Design:**

The clinical data of patients with liver failure admitted to the intensive care unit of our hospital were retrospectively analysed to identify a PE group and a sequential PE group, comprising DPMAS with PE (DPMAS + PE). The changes in liver function and coagulation function index before and after treatment and the occurrence of adverse reactions were compared.

**Results:**

In total, *n* = 57 patients (PE *n* = 14, DPMAS + PE *n* = 43) participated in the study. There was no significant difference in alanine aminotransferase (ALT), aspartate aminotransferase (AST) or total bilirubin (TBIL) levels before treatment (*p* > 0.05). After treatment, ALT levels were higher in the PE group than in the DPMAS + PE group (317.72 ± 604.24 vs. 276.02 ± 763.04, respectively), whereas TBIL levels were lower in the former than in the latter (237.91 ± 124.34 vs. 297.95 ± 153.38). The international normalised ratio (INR) was higher in the DPMAS + PE group than in the PE group (1.84 ± 0.66 vs. 1.66 ± 0.72) and prothrombin activity (30.51 ± 7.91 vs. 34.62 ± 8.43) was lower, indicating that DPMAS + PE had a better effect on ALT and AST levels, but PE alone was more effective in improving TBIL and INR levels.

**Conclusions:**

The two artificial liver treatments can improve markers of liver function in patients with liver failure and help with liver function recovery.

**Relevance to Clinical Practice:**

Both artificial liver therapies effectively support patients awaiting transplantation. Successful management, particularly of the combined DPMAS + PE modality, demands specialised nursing expertise. Nurses must vigilantly monitor circuit pressure and coagulation parameters and adjust anticoagulant doses proactively to ensure treatment safety and efficacy.

## Impact Statement

- What is known about this topic?
  ○ Liver failure is a severe condition with a high mortality rate, and liver transplantation is the ultimate treatment.
  ○ Artificial liver treatment is currently used as a bridge therapy to support patients with liver failure while awaiting transplantation.
  ○ Plasma exchange (PE) is an effective method for removing harmful substances and supplementing beneficial substances, improving liver function and survival rates.
  ○ The dual plasma molecular adsorption system (DPMAS) is a new artificial liver technology that removes bilirubin and inflammatory factors without losing autologous plasma.
  ○ Often, DPMAS needs to be used in combination with PE due to its inability to provide coagulation factors and other substances.
- What this paper adds?
  ○ This study compares the clinical efficacy and adverse reactions of PE and DPMAS + PE in patients with acute drug‐induced liver failure and provides data on the changes in liver function and coagulation function indexes before and after treatment.
  ○ In terms of efficacy, DPMAS + PE was found to be superior to PE alone in improving liver function, especially in reducing alanine aminotransferase and aspartate aminotransferase levels. However, PE alone had a better effect on improving total bilirubin and international normalised ratio levels.
  ○ Nursing observations: The study highlights the demand for specialised nursing knowledge in caring for these patients. This encompasses vigilant monitoring of the circuit and the patient, proactive prevention of complications and ensuring overall treatment safety.

## 1 Introduction

Drug‐induced liver injury (DILI) refers to severe liver impairment instigated by drugs or their metabolites. This condition leads to a substantial disruption of fundamental liver functions, including detoxification, metabolism, synthesis and biotransformation [1]. Drug‐induced liver injury manifests as a series of symptoms and diseases, such as jaundice (where the skin and sclera turn yellow due to impaired bilirubin metabolism), coagulation disorders (resulting from a deficiency in the synthesis of clotting factors by the damaged liver), hepatorenal syndrome (characterised by kidney dysfunction secondary to liver failure), hepatic encephalopathy (caused by the accumulation of toxins in the bloodstream due to the liver's inability to detoxify effectively) and ascites (which occurs when there is an abnormal buildup of fluid in the abdominal cavity) [2].

The underlying mechanisms of DILI remain incompletely understood. However, several factors are thought to play crucial roles. First, some drugs can directly damage liver cells, disrupting their normal structure and function. Additionally, an immune‐mediated response to drugs can occur, where the body's immune system mistakenly attacks the liver, leading to inflammation and cell death. Moreover, the generation of toxic metabolites during the drug metabolism process in the liver can contribute to liver injury [3]. If left untreated, DILI can have a poor prognosis, with mortality rates as high as 60%–90% [4]. Liver transplantation is often considered the ultimate treatment option for severe cases of DILI. Nevertheless, due to the critical condition of patients and the scarcity of available liver donors, the mortality rate among patients waiting for a liver transplant remains notably high.

## 2 Background

Artificial liver treatment technology has been widely used as a new treatment for patients with liver failure [5]. Research has shown that large doses of plasmapheresis therapy for patients with liver failure can significantly prolong their survival [6] without liver transplantation. The method is effective in removing harmful substances, supplementing blood coagulation factors and other beneficial substances, promoting liver cell regeneration and improving liver function, thereby improving the survival rate of patients [7]. However, in the process of artificial liver treatment, patients are highly susceptible to bleeding, hypotension, low calcium and allergic infection complications [8]. Therefore, it is necessary to monitor patients' vital signs and blood index changes closely, adjust treatment parameters accordingly and strengthen the observation of adverse reactions to ensure the safety and effectiveness of the treatment. At present, there are many specific artificial liver modes in clinical use, such as haemodialysis, haemofiltration, plasma exchange (PE), plasma perfusion, specific bilirubin adsorption, continuous blood purification and plasma dialysis filtration [9]. The dual plasma molecular adsorption system (DPMAS) is a new artificial liver technology that has become widely accepted in recent years; it can remove both bilirubin and inflammatory factors without losing autologous plasma and provide a relatively good external environment for hepatocyte regeneration [10]. However, this method cannot provide coagulation factors, albumin or other substances during the treatment process; it may even consume albumin [11] during the adsorption process and is typically used together with PE (DPMAS + PE). Few studies have compared PE alone and DPMAS + PE.

## 3 Aims and Objectives

This study retrospectively analysed the clinical data of patients with liver failure who underwent artificial liver treatment between January 2022 and December 2023. The study observed the changes in laboratory indexes of patients in the PE group and the DPMAS + PE group and calculated the adverse reactions during treatment and nursing measures to provide a reference for the observation and nursing of patients undergoing artificial liver treatment.

## 4 Design and Methods

### 4.1 Setting and Sample

A clinical analysis of patients with DILI admitted to the intensive care unit (ICU) of our hospital between January 2022 and December 2023 was performed. Before treatment, doctors usually choose between the PE regimen and the DPMAS + PE regimen based on a comprehensive consideration of factors, such as the patient's specific condition, treatment objectives, the nature of the pathogenic substances, individual differences and the preference of the patient. In this study, the included patients were divided into a DPMAS + PE group and a PE group according to the specific treatment plan adopted by clinicians. A centrifugal PE machine was used in this study. The inclusion criteria were as follows: (1) adult patients diagnosed with DILI, (2) patients meeting the diagnostic criteria for liver failure [12] (patients with prothrombin time [PT] values ≥ 40% of the standardised values or international normalised ratios [INRs] ≥ 1.5 due to severe liver damage within 8 weeks of the onset of disease symptoms are diagnosed with acute liver failure, where the liver function prior to the current onset of liver damage is estimated to be normal based on blood laboratory data and imaging examinations), (3) patients receiving artificial liver treatment during hospitalisation with PE or DPMAS + PE and (4) patients with complete clinical data. The exclusion criteria were as follows: (1) patients with other definite causes or conditions (e.g., patients who did not agree to participate in this study or had serious comorbidities, such as pulmonary infection, spontaneous peritonitis or sepsis), (2) patients with severe organ failure or systemic disease or (3) patients undergoing artificial liver therapy and those unable to complete treatment.

### 4.2 Data Collection Tools and Methods

#### 4.2.1 Data Collection

Two nurses searched the hospital's information system for patients with acute DILI who were admitted to the ICU according to the length of hospitalisation. These patients were then screened one by one according to the inclusion and exclusion criteria to identify those who met the requirements of this study. Following this, two chief nurses completed the data verification, and two hepatologists were invited to conduct the final data review. In both groups, central venous catheterisation was performed, and vascular access was checked by aspiration of the lumen before treatment. The tube was secured to the skin using the adhesive tape fixation method and punctured without bleeding. All artificial liver treatments were performed at the bedside in the ICU, with the patient's vital signs monitored by continuous electrocardiogram (ECG) monitoring and invasive blood pressure changes monitored by indwelling arterial catheterisation. In addition, clinical indicators, including blood oxygen saturation, haemoglobin, calcium, lactic acid and pH value, were monitored regularly. The treating nurse had > 5 years of ICU experience and formally signed off the artificial liver treatment independently. Patients were included for treatment once they were confirmed to have liver failure. Nurses monitored the indicators (see Section 4.2.2) before the patient's admission and discharge. For those readmitted, the measurement indicators were only adopted at the time of discharge after the second cure and formed part of the inclusion criteria for this study. During the treatment process, the occurrence time, type, severity, treatment measures and outcome of various safety events (e.g., allergic reactions and bleeding tendencies) and complications (e.g., infections and further damage to organ functions) that occurred during the treatment were systematically recorded.

#### 4.2.2 Observation Indicators

Liver function index [13]: Changes in alanine aminotransferase (ALT), aspartate aminotransferase (AST) and total bilirubin (TBIL) before and after treatment were detected using an automatic biochemical analyser.

Coagulation function index [14]: Prothrombin time, prothrombin activity (PTA) and INR were measured using an automatic coagulation analyser. Prothrombin time and INR are particularly important in assessing the extrinsic pathway of coagulation and are widely used as indicators of liver synthetic capacity.

Incidence of adverse reactions: Observations were made of the incidence of bleeding/haematoma, palpitations, hypotension and separator coagulation in the DPMAS + PE group within 72 h from the start of therapy to the end of therapy.

Bleeding/haematoma: During treatment, the occurrence of visible blood leakage at the catheter insertion site or the formation of local blood accumulation under the skin at or near the catheter insertion area was recorded, and the grade was determined. The amount of bleeding was calculated by directly measuring the volume of bleeding per unit time (mL).

Palpitations: During treatment, the time and degree of any subjective feelings of an abnormal, rapid or irregular heartbeat reported by the patient were recorded.

Hypotension: Times when the patient's systemic arterial blood pressure dropped below the normal range were recorded.

Separator coagulation: Clots formed within the blood separator component of the artificial liver support system were noted; this was confirmed by visual observation or by an increase in the blood flow resistance of the device.

#### 4.2.3 Operation Methods of Artificial Liver Treatment

The artificial liver treatment machine used for both patient groups was an IQ21 (Tianjin Hanahao Medical Materials Co. Ltd. Tianjin, China), with 5 u/mL of pre‐filled fluid and 2000 mL of replacement plasma; the initial blood flow velocity for both groups was 50 mL/min, with the blood flow velocity adjusted to the treatment level once the blood pressure was stable.

##### 4.2.3.1 The Plasma Exchange Group

Before plasma infusion, dexamethasone sodium phosphate injection (5 mg) was administered via intravenous infusion, with 10% glucose injection (10 mL) plus calcium gluconate injection (10 mL) injected intravenously to prevent anaphylactic reaction. The blood flow rate of the plasma separator was 80–150 mL/min, the plasma separation ratio was 20%–30%, the plasma separation speed was 20–30 mL/min, and the speed of replacement fluid was consistent with the plasma separation rate; 2000 mL of frozen plasma was used for each treatment, ranging from 1.0 to 1.5 h at 100–120 mL/min, with the plasma separation pump at 20%–25% and return pump at 100%.

##### 4.2.3.2 Dual Plasma Molecular Adsorption System Plus Plasma Exchange Group

Using plasma bilirubin BS330 and HA33, the total treatment volume was set to 5 L. The DPMAS treatment was followed by PE to replace 1000 mL of frozen plasma. The DPMAS treatment parameters were as follows: blood flow velocity = 100–150 mL/min, plasma ratio = 20%–30%, plasma separation velocity = 20–45 mL/min, lower limit = 1.2 × plasma volume, higher limit = 2–3 × plasma volume and treatment time = ≥ 2 h. Continuous ECG monitoring was performed, with careful observation of changes in the patient's condition.

### 4.3 Data Analysis

Data processing and analysis were performed using SPSS 22.0 statistical software. Measurement data meeting normal distribution were expressed as mean ± standard deviation and compared using the *t*‐test; those not conforming to normal distribution were expressed as the median (M [Q1, Q3]) for comparisons using the rank‐sum test. Count data were presented as percentages (%) and compared using the chi‐squared test. A *p* value of < 0.05 was considered statistically significant.

## 5 Ethical and Institutional Approvals

This study was conducted in accordance with the Declaration of Helsinki (https://www.wma.net/policies‐post/wmadeclaration‐of‐helsinki‐ethical‐principles‐for‐medical‐research‐involving‐human‐subjects/) and was approved by the Ethics Committee of our hospital (Ethics Committee File Number: LL‐2023‐166‐K, Approval Number: [2024]003, Date: 08/01/2024). Written informed consent was obtained from all participants, and written consent was also obtained retrospectively for data sharing.

## 6 Results

### 6.1 Comparison of General Data

A total of 57 patients were included. There were 14 patients in the PE group, of whom 8 were men and 6 were women, aged 20–71 years, with a mean age of 50.39 ± 15.44 years. In the DPMAS + PE group, 26 were men and 17 were women; they were aged 18–75 years, with a mean age of 49.13 ± 13.37 years. General data showed no significant difference (*p* > 0.05) (see Table 1).

**TABLE 1:** General data of the patients.

Project		PE (*n*/%)	DPMAS + PE (*n*/%)	*t*/*X* ^2^/*Z*	*p*
Sex (*n*)	Man/woman	8/6	26/17	−0.977	0.331
Age (year)	($\bar{X}\pm S$)	50.39 ± 15.44	49.13 ± 12.37	−0.359	0.72
	Hospital stays	5–25 days	5–25 days		
	Secondary hospital ratio	78%	45%		
Diagnose (*n*)	Acute hepatic failure	5 (35.71)	14 (32.56)	0.103	0.918
	Chronic liver failure	0 (0)	0 (0)		
	Subacute liver failure	4 (28.57)	10 (23.26)		
	Slow plus acute liver failure	4 (28.57)	15 (34.88)		
	Slow plus subacute liver failure	1 (7.15)	4 (9.3)		
Treatment pathway (*n*)	Inside neck tube	2 (14.29)	14 (32.56)	−1.782	0.079
	Intrafemoral vein catheterisation	12 (85.71)	29 (67.44)		
Lapse to (*n*)	Liver transplantation	3 (21.43)	9 (20.93)	0.59	0.557
	Leave hospital	8 (57.14)	24 (55.81)		
	Die	3 (21.43)	10 (23.26)		

*Note:* Slow plus acute liver failure refers to cases where liver failure progresses slowly over time but eventually reaches an acute stage, with rapid deterioration in liver function in a short period. Slow plus subacute liver failure refers to cases where liver failure progresses slowly but reaches a subacute stage, with a more gradual decline in liver function over weeks rather than days.

### 6.2 Comparison of Liver Function Indicators

There was no significant difference in ALT, AST or TBIL levels between the two groups before treatment (*p* > 0.05). After treatment, the ALT level in the PE group was higher than that in the DPMAS + PE group (317.72 ± 604.24 vs. 276.02 ± 763.04) but with no significance, whereas the TBIL level was lower in the PE group than in the DPMAS + PE group (237.91 ± 124.34 vs. 297.95 ± 153.38). The differences between the two groups were statistically significant (*p* < 0.05), indicating that the treatment adopted by the DPMAS + PE group was better in improving the liver function TBIL index. The ALT, AST and TBIL levels in the DPMAS + PE group decreased compared with before treatment (*p* < 0.05) (see Table 2).

**TABLE 2:** Comparison of laboratory indicators before and after treatment in the two groups.

Group (*n* = number of treatments)		PE group (*n* = 14)	DPMAS + PE group (*n* = 43)
ALT (U/L)	Pre‐therapy	414.05 ± 628.52	346.25 ± 795.07
	Post‐treatment	317.72 ± 604.24	276.02 ± 763.04*
AST (U/L)	Pre‐therapy	505.44 ± 1264.41	341.72 ± 686.21
	Post‐treatment	314.66 ± 771.72	233.37 ± 526.59*
TBiL (umol/L)	Pre‐therapy	379.24 ± 203.04	341.72 ± 686.21
	Post‐treatment	237.91 ± 124.34*	297.95 ± 153.38*^∆^
PT (s)	Pre‐therapy	29.67 ± 18.93	39.59 ± 22.26
	Post‐treatment	31.16 ± 9.03*	42.01 ± 17.42
PTA (%)	Pre‐therapy	21.57 ± 7.50	21.33 ± 7.21
	Post‐treatment	34.62 ± 8.43*	30.51 ± 7.91*
INR	Pre‐therapy	2.41 ± 1.04	2.14 ± 0.93
	Post‐treatment	1.66 ± 0.72*	1.84 ± 0.66*^∆^

*Note:* An asterisk (*) indicates a statistically significant difference between the post‐treatment and pre‐therapy conditions, with a *p*‐value < 0.05. A delta (∆) denotes a statistically significant difference between the PE group and the DPMAS + PE group in either the pre‐therapy or post‐treatment phase, also with a *p*‐value of < 0.05.

Abbreviations: ALT, alanine aminotransferase; AST, aspartate aminotransferase; INR, International normalised ratio; PT, test prothrombin time; PTA, prothrombin activity; TBiL, total bilirubin.

### 6.3 Comparison of Coagulation Function Indexes

There was no statistical difference in the PT, PTA or INR levels before treatment (*p* > 0.05). After treatment, the INR level in the DPMAS + PE group (1.84 ± 0.66) was higher than that in the PE group (1.66 ± 0.72); the difference between the two groups was statistically significant (*p* < 0.05). Compared with pre‐therapy, the post‐treatment PTA in the DPMAS + PE and PE groups showed statistically significant differences. This indicates that the improvement in coagulation function in the DPMAS + PE group was not as significant as that in the PE group (see Table 2).

### 6.4 Prevalence of Adverse Reactions

Adverse reactions occurred in one case in the PE group, including bleeding in the right femoral vein and large haematoma in the right thigh during treatment. There were four cases of adverse reaction in the DPMAS + PE group, including one case of subcutaneous haemorrhage after needle extraction in the right arm, one case of palpitations and two cases of hypotension. The incidence of adverse reactions was higher in the DPMAS + PE group (9.30%) than in the PE group (7.14%) (see Table 3).

**TABLE 3:** Statistics on the incidence of adverse reactions (*n*/%).

Group (*n* = number of treatments)	Bleeding/hematoma	Palpitate	Hypopiesia	Isolator clotting	Prevalence of adverse reactions
PE group (*n* = 14)	1 (7.14)	0 (0)	0 (0)	0 (0)	1 (7.14)
DPMAS + PE group (*n* = 43)	1 (2.33)	1 (2.33)	2 (4.65)	0 (0)	4 (9.30)

*Note:* Hypotension is considered low blood pressure when systolic pressure is below 90 mmHg or diastolic pressure is below 60 mmHg.

## 7 Discussion

Liver failure is a functional condition [15] caused by severe liver damage. The widespread application of artificial liver therapy provides effective treatment for patients with liver failure. The method is effective in temporarily replacing some functions of the liver, removing harmful substances in the body and improving internal circulation, thereby reducing the liver burden and providing the opportunity for patients with liver failure to transition to liver transplantation [16]. The plasma exchange mode uses a plasma separator to separate the whole blood in vitro and discard the plasma, discard the various components dissolved through the membrane pores in the plasma, retain the blood cells and platelets that cannot pass through the membrane pores and then mix the plasma with an equal amount of replacement fluid and send it back into the body [17]. The DPMAS mode draws blood out of the body and passes it through a plasma separator; the isolated plasma passes through an anionic resin plasma bilirubin adsorption column and a neutral macroporous resin adsorption column. After the bilirubin and other toxins in the plasma are absorbed, the plasma is recombined with blood cells and sent back to the body [18].

In this study, the treatment effects of PE alone and DPMAS + PE were compared, and the results showed that both methods improved liver function and promoted liver recovery. The DPMAS + PE group had stronger removal of bilirubin than the PE group, which was consistent with the results obtained by Wang et al. [10]. Due to the critical condition of patients with liver failure, various adverse reactions are liable to occur during artificial liver treatment, including haematoma or bleeding at the puncture site, palpitations, hypotension, hypercalcaemia, allergic reaction, infection, coagulation or separator membrane rupture and deep vein thrombosis of lower limbs [19]. In this study, two groups of ICU‐hospitalised patients with life‐threatening conditions underwent artificial liver treatment performed by ICU nurses at the bedside. Patients were treated by the highly trained blood purification treatment team. During treatment, patients' vital signs were monitored, with indwelling arterial catheterisation used to monitor invasive blood pressure and deep venous catheterisation used with all patients as a therapeutic pathway.

Total bilirubin reflects the detoxification function of the liver, and both AST and ALT can accurately reflect the degree of liver injury. Following treatment, the ALT level in the PE group was higher than that in the DPMAS + PE group, and the TBIL level was lower in the former than in the latter. The difference between the two groups was statistically significant, indicating that in the DPMAS + PE group, ALT, AST and TBIL decreased compared with pre‐treatment, which was similar to the study results obtained by Pan Hao et al. [20]. This indicates that artificial liver treatment improves the liver function of patients.

The liver is an important organ for maintaining the dynamic balance between the coagulation and anticoagulant systems. In patients with liver failure, the synthesis of coagulation factors decreases and the consumption is substantially increased, resulting in different degrees of coagulation disorders. As the most commonly used screening indicator reflecting the in vitro coagulation system, fibrinogen levels and coagulation factors V, VI and X play a role in determining their size, and the extension of PT is typically positively correlated with the degree of liver damage [21]. In this study, the INR was higher and the PTA lower in the DPMAS + PE group than in the PE group. This indicates that the improvement in coagulation function in the DPMAS + PE group was not as significant as that in the PE group; however, both methods help improve coagulation function. The results suggest that PE can increase the improvement in bilirubin while improving coagulation function, which is consistent with the study results obtained by Zhang Jing et al. [22].

In this study, one patient had palpitations and two patients had hypotension in the DPMAS + PE group, and neither patient group had allergies or low blood calcium manifestations. The reason for the adverse reactions may be related to the PE process following the separation and discharge of waste plasma while supplementing equal fresh plasma; after isolating plasma from the DPMAS + PE group during the early treatment for bilirubin adsorption and plasma perfusion, the blood volume was reduced, leading to the plasma colloidal venous pressure drop [23]. According to the operator of the booster drug pump, following intravenous access rehydration treatment, the patient's blood pressure gradually recovered. During treatment, attention should be paid to the pressure changes in the pipeline, and the dosage of anticoagulant drugs should be adjusted in response to increased pressure. To prevent the separator from clogging, it is advisable not to administer macromolecular drugs through the artificial liver system. If the pipeline pressure increases, the treatment should be terminated promptly to allow blood to be returned.

Effective nursing care of these patients is crucial, requiring extensive training and attention to detail to optimise patient outcomes [24]. A high‐quality nursing service system adopts a patient‐centred humanistic care model, integrating patient health needs and conditions to provide personalised and tailored quality care services [10]. To meet the complex health needs of patients and improve the quality of care, highly qualified nursing teams often set high standards for caregiver professionals [25]. This helps to enhance the professional skills and dedication of the nursing team, optimising the overall quality of care services. High‐quality nursing service systems have been shown to alleviate psychological distress in patients while maintaining high efficacy and safety. In this study, two specialist nurses provided effective nursing care for the patients.

### 7.1 Limitations

This study has some limitations. First, only patients with liver failure from one hospital were selected for retrospective observation, and the sample size of the PE treatment group was small; therefore, the statistical results may have some bias. In the future, the sample size should be expanded for prospective observational studies to further establish the advantages of DPMAS + PE treatment and provide evidence for the treatment of patients with clinical liver failure. Additionally, different analytical methods, such as multivariate analysis, should be used for a more comprehensive data analysis.

## 8 Recommendations or Implications for Practice and Further Research

This small retrospective observation study suggests that the DPMAS + PE treatment may be superior to PE alone in improving liver function. To improve the coagulation effect of DPMAS + PE treatment, timely plasma supplementation, optimisation of the treatment protocol or the addition of adjunctive therapies could be considered to better meet the individual needs of patients.

## 9 Conclusion

Both methods of artificial liver treatment assessed in this study improve the liver function of patients and provide opportunities for those waiting for liver transplantation. This observational study demonstrates that in terms of improving liver function, DPMAS + PE treatment presents a better trend than PE treatment alone, but its effect on improving coagulation function may not be as good as that of PE treatment alone. The specific treatment plan should be selected based on the characteristics of the patient. The incidence of adverse reactions in the DPMAS + PE group was relatively high in the observation, which might be related to the longer treatment duration and the failure to replenish plasma colloid osmotic pressure in time at the beginning of treatment. Both treatment processes require close observation throughout. High‐quality care helps ensure the smooth progress of the treatment and improve the therapeutic effect.

## Funding

This work was supported by Beijing You'an Hospital Affiliated to Capital Medical University 2023 Incubation Project for Young and Middle‐aged Talents in the Hospital (Special Construction Project–Nursing Research) (No.: BJYAYY‐YN2023‐23), Research on the Transformation and Application of Electrocardiogram Monitoring Output Line Receiver (BJYAYY‐YN2023‐34), Based on the concept of “adjusting the body, regulating the breath and regulating the mind”, this paper explores the intervention effect of sitting Baduanjin on elderly patients at high risk of sarcopenia complicated with HIV/AIDS (BJYAYY‐YN2023‐29), and The application of the “Internet Plus” hospital‐family integrated model in the continuous alcohol abasment management of alcoholic liver disease patients with alcohol dependence (BJYAYY‐YN2023‐26).

## Ethics Statement

This study was conducted in accordance with the Declaration of Helsinki (https://www.wma.net/policies‐post/wmadeclaration‐of‐helsinki‐ethical‐principles‐for‐medical‐research‐involving‐human‐subjects/) and approved by the ethics committee of Beijing You'an Hospital (Ethics Committee File Number: LL‐2023‐166‐K, Approval Number [2024]003, Date: 08/01/2024).

## Consent

Written informed consent was then obtained from all participants.

## Conflicts of Interest

The authors declare no conflicts of interest.

## Data Availability

The authors confirm that the data supporting the findings of this study are available within the article.
